# The Regional EEG Pattern of the Sleep Onset Process in Older Adults

**DOI:** 10.3390/brainsci11101261

**Published:** 2021-09-24

**Authors:** Maurizio Gorgoni, Serena Scarpelli, Ludovica Annarumma, Aurora D’Atri, Valentina Alfonsi, Michele Ferrara, Luigi De Gennaro

**Affiliations:** 1 Department of Psychology, Sapienza University of Rome, 00185 Rome, Italy; serena.scarpelli@uniroma1.it (S.S.); valentina.alfonsi@uniroma1.it (V.A.); luigi.degennaro@uniroma1.it (L.D.G.); 2 Body and Action Lab, IRCCS Fondazione Santa Lucia, 00179 Rome, Italy; ludovica.annarumma@uniroma1.it; 3 Department of Biotechnological and Applied Clinical Sciences, University of L’Aquila, 67100 L’Aquila, Italy; aurora.datri@univaq.it (A.D.); michele.ferrara@univaq.it (M.F.)

**Keywords:** sleep onset, wake-sleep transition, EEG topography, slow wave activity, aging, EEG power, NREM sleep

## Abstract

Healthy aging is characterized by macrostructural sleep changes and alterations of regional electroencephalographic (EEG) sleep features. However, the spatiotemporal EEG pattern of the wake-sleep transition has never been described in the elderly. The present study aimed to assess the topographical and temporal features of the EEG during the sleep onset (SO) in a group of 36 older participants (59–81 years). The topography of the 1 Hz bins’ EEG power and the time course of the EEG frequency bands were assessed. Moreover, we compared the delta activity and delta/beta ratio between the older participants and a group of young adults. The results point to several peculiarities in the elderly: (a) the generalized post-SO power increase in the slowest frequencies did not include the 7 Hz bin; (b) the alpha power revealed a frequency-specific pattern of post-SO modifications; (c) the sigma activity exhibited only a slight post-SO increase, and its highest bins showed a frontotemporal power decrease. Older adults showed a generalized reduction of delta power and delta/beta ratio in both pre- and post-SO intervals compared to young adults. From a clinical standpoint, the regional EEG activity may represent a target for brain stimulation techniques to reduce SO latency and sleep fragmentation.

## 1. Introduction

In the last decades, research has questioned the “classic” view of sleep and wakefulness as discrete and mutually exclusive states, describing the co-existence of regional electrophysiological markers of sleep and wakefulness in different cerebral areas [1,2]. In this view, sleep should be considered as a locally regulated process [3,4].

Healthy physiological aging is characterized by relevant modifications of the sleep pattern, mainly characterized by longer sleep onset (SO) latency, decreased sleep duration, greater sleep fragmentation and time spent awake after the beginning of sleep, and reduced slow wave sleep (SWS) (for a review, see [5]). A growing body of evidence suggests that regional electroencephalographic (EEG) sleep features also undergo wide changes during healthy aging [5]. In particular, older adults exhibit decreased oscillatory activity during non-REM (NREM) sleep in different frequency ranges [6]. The reductions of slow wave (0.5–4.5 Hz) and spindle (12–16 Hz) activity represent the greatest age-related modifications, mainly observable in the frontal region [6,7,8,9,10,11,12,13]. The age-related alterations in such regional sleep EEG hallmarks are associated with impaired cognitive functioning (i.e., memory processes) and brain integrity [10,11]. In normal conditions, the slow wave activity (SWA) exhibits a homeostatic regulation: during wakefulness, it increases before the SO and after sleep deprivation; during sleep, it characterizes the NREM stage, reaching its maximum during the beginning of the sleep episode and showing a progressive reduction during the night [1,2]. An impairment of the homeostatic regulation process has been observed in older compared to young adults [8,14,15,16]. Moreover, the phase-locked synchrony between sleep spindles and slow waves also appears altered in the elderly [17,18]. During REM sleep, a decreased power in delta, theta, and alpha frequencies has been found, mainly expressed in the central region [8,19].

In this field of knowledge, one of the missing pieces is represented by the effect of physiological aging on the spatiotemporal EEG dynamics of the sleep onset (SO), the complex and progressive transitory process between wakefulness and sleep [20,21]. From an electrophysiological standpoint, the wake-sleep transition in healthy young adults is characterized by extensive modifications [22,23], mainly consisting of (a) an orchestrated pattern of regional and gradual frequency-specific changes observable from the scalp EEG recordings [24,25,26,27], (b) asynchronies in the EEG pattern of cortical and deep brain structures [28,29], and (c) marked changes in cerebral dynamic interactions [30,31,32,33]. Moreover, the electrophysiology of the falling asleep process is modulated by the level of homeostatic sleep pressure [33,34,35,36,37]. A recent high-density EEG study found a different spatiotemporal pattern of the slow waves’ regulation during the wake-sleep transition in children compared to young adults [38], providing evidence for the influence of a developmental factor on the regional electrophysiology of the SO. According to this notion and considering (a) the longer sleep latency that characterizes SO in the elderly [5], (b) the age-related regional EEG sleep modifications [5], and (c) the evidence of impaired homeostatic regulation of the SWA in the elderly [8,14,15,16], it could be hypothesized that there is an influence of healthy aging on the regional and temporal EEG pattern of the falling-asleep process.

The aim of the present study is to describe for the first time the regional EEG features of the wake-sleep transition in a group of healthy older adults, assessing the spatial changes in the EEG power spectra that characterize the transition from wakefulness to sleep using 1 Hz bins resolution in the 0.5–24 Hz range, and the time course of the topographical EEG frequency bands during the SO. Moreover, starting from the observed maturational changes in the slow wave regulation [38], we provide a direct comparison between the elderly subjects and a group of young adults, performed on the regional pattern of the delta EEG power. Finally, since the longer SO latency observed in the elderly may be related to a greater level of psychophysiological arousal, we analyzed an electrophysiological index of activation, the delta/beta ratio [39,40], which represents a reliable indirect measure for the assessment of the arousal level [41,42]. Specifically, we compared the delta/beta ratio between older and young adults.

## 2. Materials and Methods

### 2.1. Participants

Forty healthy older volunteers were recruited from local senior centers in Rome. Four subjects were excluded from the analyses after visual inspection of the polysomnographic (PSG) recordings because they did not exhibit artifact-free epochs in the pre-SO interval. Therefore, the analyses were performed on 36 subjects (16 F; mean age ± SE: 68.4 ± 1.08 years; age range: 59–81 years). The inclusion criteria were as follows: absence of psychiatric or neurological disorders and cognitive impairment; absence of sleep disturbances and/or the condition of excessive daytime sleepiness (EDS); absence of other relevant medical conditions and obesity; no use of psychoactive, hypnotic drugs, or relevant medication that might affect the sleep EEG; absence of alcohol and other substance addiction; and regular sleep-wake rhythms. A clinical and anamnestic interview was administered during the recruitment phase to determine the eligibility for the enrolment in the study. All older adults underwent cognitive screening through the Mini Mental State Examination (MMSE) (mean corrected MMSE ± SE: 27.04 ± 0.24), and subjective sleep quality was assessed by the Italian version of the Pittsburg Sleep Quality Index (PSQI) (mean PSQI ± SE: 6.58 ± 0.62). 

All subjects gave their written informed consent. The study was approved by the Institutional Ethics Committee of the Department of Psychology of the “Sapienza” University of Rome (#1128/2016; 1 December 2016) and was conducted in accordance with the Declaration of Helsinki.

For the specific analyses performed on relative SWA power and delta/beta ratio (see below), we performed a direct comparison between the elderly subjects and a group of 40 healthy young adults (20 F, mean age: 23.8 ± 2.88 years; age range: 18–29 years) from the database of our laboratory, recorded with the same electrodes montage. The spatiotemporal EEG pattern during the SO process in this group of young adults has been previously described (see [26] for PSG recordings information, methodological details, and results). The inclusion criteria were as follows: normal sleep duration and schedule; no daytime nap habits; no EDS; no other sleep, medical, neurologic, or psychiatric disorders, assessed by a 1-week sleep log and a clinical interview; and no use of psychoactive, hypnotic drugs, or relevant medication that might affect the sleep EEG.

### 2.2. Study Design

Elderly participants were requested to respect a regular sleep-wake rhythm during the week before the experimental session and fill a sleep log each morning to control their compliance.

On the day of the experimental session, each participant came to the Sleep Psychophysiology Laboratory of the “Sapienza” University of Rome at 8.00 p.m. and underwent electrodes montage (~2 h). The PSG recordings were performed in a sound-proof, temperature-controlled room. The subject’s sleep was undisturbed, started according to the individual habitual sleep schedule. PSG recordings ended after the final morning spontaneous awakening.

Concerning the previously recorded young adults, the recording conditions were the same of the elderly participants, with the only exception that the analyzed PSG data were referred to a baseline recording night following to an adaptation night in the sleep laboratory, and followed by a protocol of 40 h sleep deprivation with a subsequent recovery night.

### 2.3. Polysomnographic Recordings

A Micromed system plus digital polygraph was used for PSG recordings of the elderly subjects. PSG signals were acquired with a sampling rate of 256 Hz, A/D converted at 16 bit, and bandpass pre-filtered (hardware input filter) at 0.5–40 Hz. A notch filter (50 Hz) was also applied. The EEG signals of the 19 unipolar derivations of the international 10–20 system (Fp1, Fp2, F3, F4, F7, F8, Fz, C3, C4, Cz, P3, P4, Pz, O1, O2, T3, T4, T5, T6) were acquired using Ag/AgCl electrodes, referenced to the ground electrode (Fpz) and offline re-referenced to the averaged mastoids (A1–A2). Electrooculogram (EOG) electrodes were placed ~1 cm from the medial and lateral canthi of the dominant eye. Submental electromyogram (EMG) signals were also recorded. Impedances were kept below 5 KΩ. A pulse oximeter placed on the right index finger was used to monitor oxygen saturation to exclude sleep-breathing disorders.

PSG signals of the young adults were recorded by an Esaote Biomedica VEGA 24 polygraph using the same montage of the elderly adults with average mastoid reference.

### 2.4. Data Analyses

#### 2.4.1. PSG Measures 

Sleep scoring and artifact rejection were manually performed by and expert sleep researcher. The recorded EEG derivations, EMG, and EOG were used for the visual scoring of the sleep stages in 12 s epochs according to the standard criteria [43]. Epochs containing ocular and muscular artifacts [43] were manually rejected. Slow wave sleep (SWS) scoring strictly fulfilled the >75 μV amplitude criterion. The following variables were assessed: stage 1 and stage 2 latency in minutes; total sleep time (TST), defined as the sum of time spent in stage 1, stage 2, SWS, and REM; percentage of each sleep stage (time spent in a sleep stage/TST × 100); wakefulness after sleep onset (WASO), expressed in minutes; number of awakenings; number of arousals; total bed time (TBT); and sleep efficiency index (SEI = TST/TBT × 100). An awakening was scored whenever EEG/EMG activation occurred lasting >10 s. Arousals were scored whenever EMG activation affected the EEG recording for periods <10 s. PSG measures of the elderly and young groups were compared using unpaired t-tailed *t*-tests. 

#### 2.4.2. Quantitative Analyses of the EEG Signals

We considered the 0.50 to 25.00 Hz frequency range, computing power spectra (fast Fourier transform routine) for 4 s periodograms. Spectra from three consecutive 4 s epochs were averaged to allow for alignment with the visual scoring of sleep stages, based on 12 s epochs. The individual SO was defined based on the appearance of the first K-complex or sleep spindle [26,36]. We assessed the EEG topography during the 5 min pre-SO and post-SO. Ocular and muscular artifacts were excluded after visual inspection. Data analyses were performed using MATLAB R2011b.

#### 2.4.3. Single Hz EEG Topography

Data were reduced to a 1 Hz bin width by collapsing four contiguous 0.25 Hz bins before performing the statistical analyses, with the only exception of the 0.5 to 1 Hz bin. In this case, two adjacent 0.25 Hz bins were collapsed. The log-transformed EEG power values for each 1 Hz bin were considered dependent variables and compared (paired *t*-tests) in the 5 min intervals before and after SO in each cortical derivation. The α-values of the comparisons were corrected by false discovery rate (FDR, [44]). 

#### 2.4.4. Time Course of the EEG Frequency Bands

With the aim to make the individual time courses comparable in the group of older adults, we performed the same procedure previously used to describe the time course of the SO process in healthy young adults [26,36]: (a) the individual time courses were aligned as a function of the first spindle/K-complex; (b) the time series of 12 s epochs of the pre-SO period were divided into five segments, and the time series of the first NREM sleep cycle were divided into 20 segments (percentiles); (c) we removed epochs with muscle or ocular artifacts and averaged individual time courses across subjects. With this method, each pre- and post-SO interval represented a 5th and a 20th percentile of the overall pre- and post-SO intervals, respectively. No SO REM sleep episodes were observed, while only two participants skipped the first REM sleep cycle.

The EEG power maps for the five time intervals before and after SO were computed in the following frequency bands: delta (0.5–4.75 Hz), theta (5.00–7.75 Hz), alpha (8.00–11.75 Hz), sigma (12.00–15.75 Hz), and beta (16.00–24.75 Hz). Moreover, power maps for the post-SO 10th, 15th, and 20th segments were computed, providing a synoptic description of the topographical EEG kinetics during the first sleep cycle. 

The overall mean duration ± standard error (SE) was 17.25 ± 2.98 min for the pre-SO period and 80.72 ± 5.73 min for the first sleep cycle. The mean duration of the artifact-free period was 5.38 ± 0.79 min for the pre-SO and 57.45 ± 3.71 min for the first sleep cycle.

#### 2.4.5. Comparison between Young Adults and Older Subjects

The direct comparisons between the elderly participants and the group of previously recorded young adults were performed on two specific measures:

(a) SWA: It represents the most intense modification in the human EEG across the wake-sleep transition [26,36]. A maturational influence of the regulation of the slow waves during the SO has been recently observed comparing children and adults [38]. With the aim to minimize possible between-group differences associated with non-specific factors (e.g., skull thickness) and to reduce between-subject variability, the spectral power of the 0.25 Hz bins in the delta frequency range (0.5–4.75 Hz) were expressed as the percentage of the total power spectrum of each cortical derivation, and then summed together to obtain the relative EEG power of the delta band. Such procedure was performed separately for each time interval (pre- and post-SO) and age group (young and older adults);

(b) delta/beta ratio: It represents a reliable index of the arousal level [41,42]. For each scalp location, we computed such integrated EEG index of activation [39,40]. 

For both relative delta power and delta/beta ratio, we performed a two-way mixed ANOVA on the relative delta power for each scalp derivation, with age (young vs. older adults) as the between-subject factor and time (pre- vs. post-SO) as the within-subject factor. With the aim to illustrate the topography of the ANOVAs’ main effects accounting for their directions, we computed the *t*-value corresponding to each *F*-value [45]. The sign of each *t*-value was defined by the difference between the mean values of the levels of each factor. The FDR was applied to correct the α-value. Two-tails *t*-tests were performed for post hoc comparisons in case of significant interactions.

## 3. Results

### 3.1. PSG Measures

PSG measures are reported in Table 1, describing in older adults a normal night of sleep expressed by substantially similar values compared with our previous studies [19,46,47,48,49]. The direct comparison between the two age groups highlighted a reduced sleep quality and duration in the elderly. Specifically, compared to young adults, the older ones exhibited longer stage 1 latency, higher percentage of stage 2, reduced percentage of SWS and REM sleep, and reduced number of awakenings and arousals, but greater overall WASO and lower TBT, TST, and SEI. 

### 3.2. Scalp Topography of the Single Hz EEG

**Figure 1:**
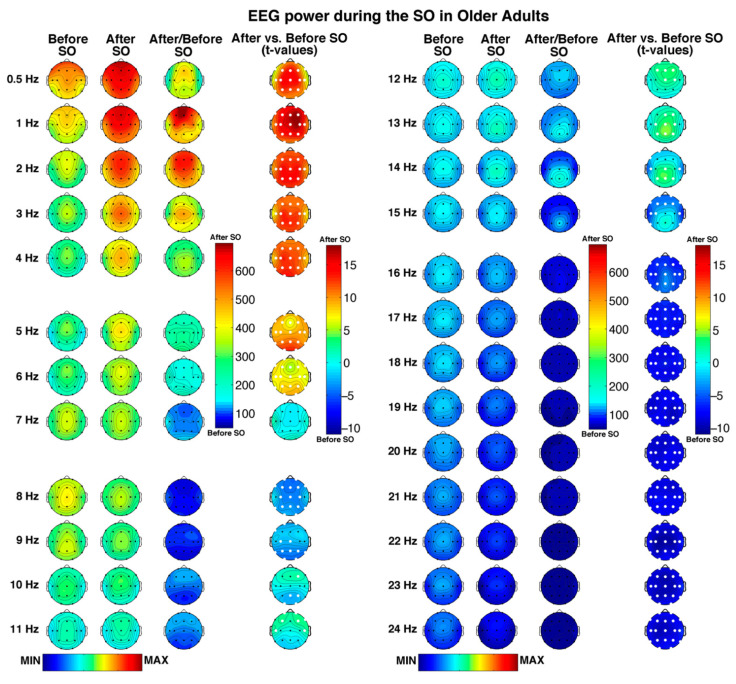
Single Hz electroencephalographic (EEG) topography (left side, frequency range: 0.5–11 Hz; right side, frequency range: 12–24 Hz) during the SO process in older adults. In both halves of the figure, the first two columns depict the topographic distribution of log-transformed absolute EEG power in the 5 min intervals before and after SO, respectively. The maps were scaled between minimal (min) and maximal (max) values calculated for all frequencies and derivations in pre- and post-SO periods. The third column shows the relative EEG changes expressed as the ratio between after and before SO period (Post-SO/Pre-SO × 100). The maps were scaled between min and max values calculated for all frequencies and derivations. The fourth column illustrates the topographical statistical EEG power differences (assessed by paired *t*-tests) between the post- and pre-SO periods. Values are expressed in *t*-values: positive *t*-values indicate a prevalence of the after SO period and vice versa. White dots indicate significant differences after the FDR correction (critic *p* = 0.037). Values are color-coded and plotted at the corresponding position on the planar projection of the scalp surface and are interpolated (biharmonic spline) between electrodes. The maps are based on the 19 unipolar EEG derivations of the international 10–20 system with averaged mastoid reference, and they are plotted for each frequency Hz bin in the 0.50–24.75 Hz range.

Figure 1 depicts the regional features of the pre- and post-SO periods in older adults with a 1 Hz resolution, as well as their comparison (i.e., ratios and *t*-tests), showing a large number of significant differences (critic *p* after FDR correction = 0.037) between the considered time intervals. 

A generalized spectral power increase after SO, involving all scalp derivations, can be observed in all frequency bins ≤6 Hz. In both pre- and post-SO period, the slowest frequency bins in the delta range showed a clear frontal maximum, becoming progressively more central in the fastest delta bins (i.e., 3–4 Hz). Moreover, the overall post-SO increase exhibited anterior and posterior maxima in the slowest and fastest delta frequency bins, respectively.

Bins in the theta band were characterized by a frontocentral maximum across the whole SO process, with a global post-SO increase at 5–6 Hz that reached its peak in the occipital region. No pre- vs. post-SO significant changes in the 7 Hz bin were observed.

Frequencies in the alpha range exhibited a bin-specific pattern of SO-changes. In the 8 Hz bin, characterized by an overall central predominance, we found a generalized decrease after SO (excluding two bilateral temporal derivations). The 9–11 Hz bins exhibited a shift from a pre-SO posterior to a post-SO anterior predominance but showed frequency-specific significant changes: (a) a reduction in posterior, central, and frontopolar derivations at 9 Hz; (b) a posterior decrease, and an increase in a single right frontal location at 10 Hz; and (c) a wide anterior increase at 11 Hz.

While the topography of the spectral power in the sigma range is characterized by a centroparietal maximum, particularly evident after the beginning of sleep, the pre- vs. post-SO comparison points to a bin-specific pattern of changes, including the following: (a) a frontocentral increase at 12 Hz, which became more posterior at 13–14 Hz, reaching its peak in the centroparietal areas; (b) a reduction at 14 Hz involving two bilateral temporal locations and two derivations in the left frontal-frontopolar regions, which became more widely distributed in the frontotemporal areas at 15 Hz.

Finally, bins in the beta frequency (16–24 Hz), mainly characterized by midline central maxima, exhibited a global post-SO decrease, which involved all cortical derivations.

### 3.3. Time Course of the EEG Frequency Bands

**Figure 2:**
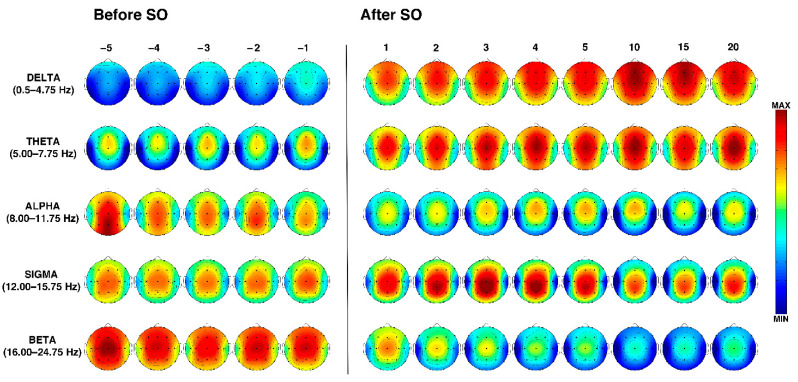
Time course of EEG frequency bands during the SO process in older adults. From the left, topographic EEG shows changes across the interval preceding SO, that is, from the lights off to the first epoch of stage 2 NREM sleep. The power maps were obtained by subdividing the pre-SO interval into five equal parts (from the –5th to –1st). Data were calculated for each subject and then averaged across subjects. After the vertical line, indicating the first epoch of stage 2 sleep, the first five intervals after SO (from the 1st to the 5th) are plotted. The last three columns on the right side show power maps at the 10th, 15th, and 20th time intervals calculated across the first sleep cycle. Values are color-coded and plotted at the corresponding position on the planar projection of the scalp surface and are interpolated (biharmonic spline) between electrodes. The maps are based on the 19 unipolar EEG derivations of the international 10–20 system with averaged mastoid reference. Maps are plotted for the following EEG bands: delta (0.50–4.75 Hz), theta (5.00–7.75 Hz), alpha (8.00–11.75 Hz), sigma (12.00–15.75 Hz), and beta (16.00–24.75 Hz).

The temporal dynamics of the EEG topographical modifications across the wake-sleep transition and during the first sleep cycle in the elderly are depicted in Figure 2.

During the entire SO period, delta activity showed an anterior predominance, and a progressive power enhancement began before SO and became larger after SO with an antero-posterior gradient. The highest peaks can be observed in the midsagittal frontal location, which showed a reduction only in the last time interval (i.e., in close proximity to the first REM episode).

Theta power was characterized by a stable frontocentral predominance across the entire wake-sleep transition, which showed a progressive increase after SO, also encompassing fronto-polar and parieto-occipital derivations. A slight midline frontocentral decrease can be observed during the first NREM sleep cycle in the 15th percentile, followed by an increase immediately before the beginning of REM sleep (i.e., the 20th percentile).

At the beginning of the pre-SO period, alpha power was characterized by a parieto-occipital prevalence (which represents the highest alpha power detected during the falling asleep process) that gradually decreases. Compared to the pre-SO intervals, the post-SO period was characterized by a more anterior pattern (with peaks over the midline frontocentral locations) and a strong power reduction in the posterior derivations.

Sigma power exhibits a quite stable central prevalence during the entire pre-SO period. After SO, it clearly increases with centroparietal maxima, exhibiting a progressive enhancement from the 1st to the 4th post-SO intervals, followed by a slight reduction.

Finally, the spectral power in the beta band revealed a midline central maximum during the whole wake-sleep transition and a widespread progressive reduction after SO.

### 3.4. Age-Dependent Modifications in SWA and Delta/Beta Ratio

#### 3.4.1. Relative Delta Power

**Figure 3:**
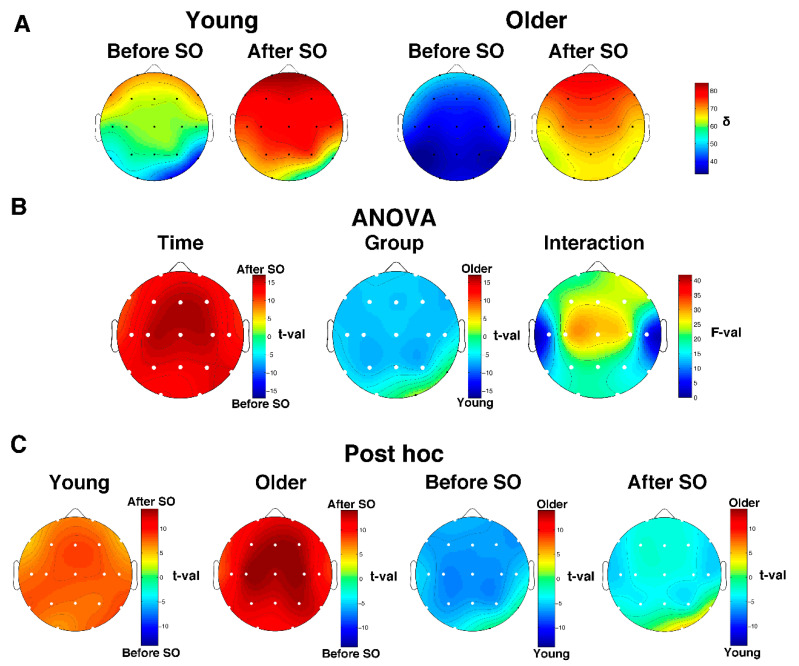
Relative SWA (0.5–4.75 Hz) power in young and older adults. (**A**) Topographical scalp maps of the SWA in the 5 min intervals before and after SO in young and older adults. The maps were scaled between minimal and maximal values calculated for all the derivations in pre- and post-SO periods in both age groups. (**B**) The results of the two-way mixed ANOVA on SWA values for each scalp derivation, with age (young vs. older adults) as the between-subject factor and time (before vs. after SO) as the within-subject factor. Results for the main effects of Group and Time are expressed in *t*-values corresponding to the original *F*-values [49], aiming to highlight the direction of differences. The sign of each *t*-value was defined by the difference between the mean values of the levels of each factor. Results for the effect of the interaction are expressed in *F*-values. White dots indicate significant differences after the FDR correction (critic *p* = 0.018). (**C**) Post hoc *t*-tests. White dots indicate significant differences.

The descriptive scalp maps of the relative SWA power (Figure 3A) showed a clear anterior prevalence in the pre- and post-SO period in both young and older subjects. Results of the two-way mixed-design ANOVAs Time x Group performed on SWA values (Figure 3B) showed a significant (critic *p* after FDR correction = 0.018) main effect of Time in all cortical derivations and Group in almost all cortical derivations (i.e., except for O2 and T6). The Time effect points to a topographically global increase of SWA after SO, while the Group effect is characterized by a generalized SWA reduction in the elderly compared to the young adults. We found a significant interaction in all cortical derivations. Post hoc *t*-tests (Figure 3B) show that both young and older adults exhibited a post-SO increase of relative delta power that involved the entire cortical topography; on the other hand, in both pre- and post-SO intervals, older adults were characterized by a lower relative delta activity compared to young adults, except for the right occipitotemporal derivations, which showed no between-group difference in the pre-SO period and higher relative delta power in the elderly after SO.

#### 3.4.2. Delta/Beta Ratio

**Figure 4:**
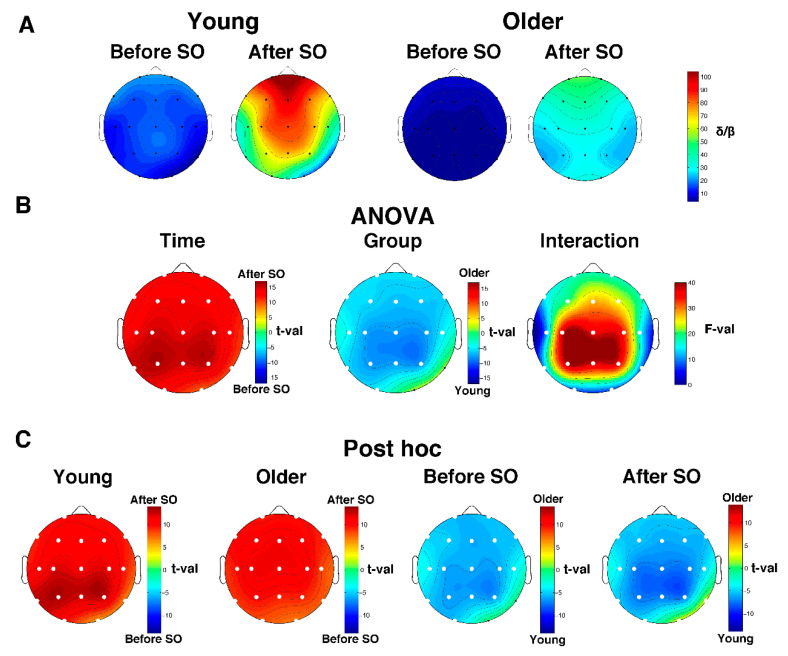
Delta/beta ratio in young and older adults. (**A**) Topographical scalp maps of the Delta/beta ratio in the 5-min intervals before and after SO in both young and older adults. The maps were scaled between minimal and maximal values calculated for all the derivations in pre- and post-SO periods in both age groups. (**B**) Results of the two-way mixed ANOVA on the delta/beta ratio for each scalp derivation, with age (young vs. older adults) as between-subject factor and time (before vs. after SO) as within-subject factor. In this case, the results for the main effects of Group and Time are expressed in *t*-values corresponding to original *F*-values, aiming to highlight the direction of differences. The sign of each *t*-value was defined by the difference between the mean values of the levels of each factor. Results for the effect of the interaction are expressed in *F*-values. White dots indicate significant differences after the FDR correction (critic *p* = 0.0073). (**C**) Post hoc *t*-tests. White dots indicate significant differences.

The analysis on the delta/beta ratio substantially mirrors the one performed on relative SWA. Both groups exhibited an anterior prevalence of the delta/beta ratio during the whole wake-sleep transition (Figure 4A). The two-way mixed-design ANOVAs *Time x Group* (Figure 4B) showed a significant (critic *p* after FDR correction = 0.0073) main effect of Time (all derivations) and Group (all derivations except for O2 and T6), as well as a significant interaction in the whole scalp topography. Specifically, the topography of the Time effect pointed to a global post-SO increase of the delta/beta ratio, while the Group effect is characterized by a lower delta/beta power in the older compared to the young adults, encompassing the whole scalp derivation with the exception of O2 and T6. Post hoc comparisons (Figure 4C) depicted a generalized increase of the delta/beta ratio after SO in both age groups. Moreover, the older subjects exhibited a reduced delta/beta ratio compared to the young adults in both pre- and post-SO. The only exception is represented by the right occipitotemporal derivations: no between-group difference (before SO) or greater delta/beta ratio in the elderly (after SO) was observed.

## 4. Discussion

To the best of our knowledge, this is the first complete description of the spatiotemporal EEG pattern during the SO in a group of older adults. Beyond confirming the main electrophysiological features previously observed in the wake-sleep transition of young adults [22,23], we found several peculiarities in the elderly: (a) the generalized post-SO power increase in the slowest frequencies (i.e., delta–theta range) did not encompass the 7 Hz bin; (b) alpha power revealed a complex frequency-specific pattern of significant post-SO modifications; (c) sigma activity exhibited only a slight post-SO increase, and the highest bins in this frequency range also showed a frontotemporal power decrease.

The direct comparison between young and older adults showed a generalized reduction of relative delta power and delta/beta ratio in both pre- and post-SO intervals in the elderly, with the only exception of the right occipitotemporal locations that exhibited no between-group difference in the pre-SO period and increased SWA and delta/beta ratio in the elderly. 

### 4.1. SWA and Delta/Beta Ratio

SWA in the elderly substantially replicates the spatiotemporal pattern previously observed in young adults [26], showing the following: (a) a frontocentral predominance (more anterior for the “slowest” bins; more central for the “fastest” bins); (b) an intense and generalized post-SO power increase, observable in both 1 Hz bin absolute power and overall relative SWA power analyses; and (c) the earliest increase observable in the frontal region, which began before SO and progressively spread with an anteroposterior gradient. The topography of these phenomena mirrors the one observed in young adults, being more prominent and earlier in the anterior regions [24,25,26,27,50,51], confirming the higher sleep need of the anterior part of the cortex. These results suggest that in older adults the build-up of the SWA also represents the strongest and early EEG phenomenon that characterizes the transition to sleep [24,25,26]. Moreover, we also found a generalized delta/beta ratio increase after SO in both young and older adults, pointing to a strong SO-related reduction of the arousal level in both age groups. On the other hand, we observed a global reduction of SWA and delta/beta ratio in older compared to young adults in both pre- and post-SO intervals. This finding is consistent with the well-described aging-related reduction of SWA [6,7,8,9,10], which mainly interests the first NREM cycles [6,7,8,9,10], extending such evidence to the wake-sleep transition. Therefore, while the spatiotemporal pattern of SWA regulation during the wake-sleep transition is substantially unchanged with increasing age, the ability of an older brain to produce sleep slow waves appears impaired across the entire SO process. Slow waves exhibit a reduction of amplitude and density with age [52,53], and their steepness appears shallower [52]. Therefore, our findings concerning SWA may be related to quantitative and/or morphological age-related changes in the slow waves. Moreover, it is worth noting that the homeostatic regulation process of the sleep SWA appears impaired in older compared to young adults [8,14,15,16]. In this view, the reduced SWA at the beginning of sleep may indirectly represent a sign of age-related reduction of the homeostatic dynamic of the SWA.

The generalized reduction of the delta/beta ratio in both pre- and post-SO in the elderly represents an index of increased arousal level during the falling asleep process. Indeed, a low delta/beta ratio is considered an indicator of worse sleep quality, and it is associated with sleep complaints in insomnia patients [41]. Subjects with primary insomnia showed a lower delta/beta ratio during the SO period than healthy controls [42]. In this view, our finding may represent the electrophysiological basis underlying the greater SO latency and sleep fragmentation that characterize older adults [5]: a greater arousal level expressed at an electrophysiological level may underlie the increased difficulty to begin sleep and maintain it during its initial phase in the elderly. 

The right occipitotemporal area represents the only exception to this pattern, showing for both SWA and the delta/beta ratio no age-related differences in the pre-SO period and an increase in the post-SO period. The possible functional meaning of this regional specificity needs further investigation. It is worth noting that Siclari and coworkers [27] described two types of slow waves during the SO process with distinct morphological and spatiotemporal patterns. Interestingly, such temporally dissociated slow wave synchronization processes have been not observed in children [38]. In this view, and starting from our present results, future studies should be directly focused on the assessment of possible differential aging-related spatiotemporal patterns of the two slow wave types. Also, the topography of the genuine oscillatory EEG activity should be evaluated. 

### 4.2. Theta Activity

Theta activity in the elderly mainly exhibits the same SO-related features observed in young adults [26]: (a) a frontocentral maximum in both pre- and post-SO; (b) a wide post-SO power increase, with maxima observed in the occipital derivations; (c) the post-SO strengthening of theta activity is progressive, spreading from the frontocentral area to the posterior regions. The only difference from the pattern previously described in young adults [26] is represented by the absence of a post-SO theta increase in the 7 Hz bin in the elderly. An age-related reduction in theta activity during NREM sleep has been previously observed [6,14]. Therefore, the absence of an increase in the 7 Hz range after the SO may represent an indirect index of the reduced theta power during NREM sleep in this population. On the other hand, the 7 Hz bin represents the border between theta and alpha activity. Considering the peculiar post-SO power reduction observed at 8 Hz (see below), the absence of difference at 7 Hz may be caused by a slowing of the alpha rhythm.

### 4.3. Alpha Activity

In young adults, alpha power exhibits a specific pattern during the wake-sleep transition [22,23]: before SO, it is characterized by an occipital maximum that gradually disappears; after SO, it progressively increases, with maximum values observed in the frontocentral regions [25,26,54,55]. Comparing the 5-min before and after-SO, we previously found a significant post-SO frontocentral increase at 8 Hz and 11 Hz [26]. Such pattern of EEG changes can be interpreted as a sign of a different functional meaning of the alpha activity before and after SO [30,56]: an occipital “idle” rhythm before SO; a component of the frontocentral synchronization process with a role in sleep-maintaining mechanisms after SO. 

Our results in older adults depict a more complex, bin-specific pattern. On the one hand, we confirmed in the elderly the anterior post-SO increase at 11 Hz, extended to a single right frontal derivation at 10 Hz, suggesting that the anterior high-alpha activity maintains a role in the overall process of synchronization during healthy aging. Moreover, the time course of the alpha power described the classical progressive posterior pre-SO prevalence, which progressively disappears, being replaced by a frontocentral predominance after SO. On the other hand, we found a reduced power after SO at 8 Hz (widely distributed on the scalp), 9 Hz (in central-posterior and frontopolar locations), and 10 Hz (in parieto-occipital regions). The posterior reduction in the 8–10 Hz range is consistent with the view of a disappearance of the occipital alpha peak (i.e., the “idle” rhythm) characterizing the wake-sleep transition [26]. At the same time, the power decrease after SO in anterior locations observed at 8–9 Hz is at odds with results in young adults. A reduction in alpha power during NREM has been previously observed in older adults [14], and was more pronounced in the frontocentral derivations in the high-alpha frequencies. On the other hand, Sprecher and coworkers [6] did not find age-related changes in the alpha range. During wakefulness, the alpha rhythm exhibits the widest age-related EEG modification, mainly represented by a strong decrease in the alpha amplitude and a slowing of the background dominant alpha rhythm [57,58,59]. Babiloni and coworkers [60] observed a reduced magnitude of the posterior alpha rhythm during aging, associated with the global cognitive level. The posterior alpha reduction was observed in both high-alpha (10–13 Hz) and low-alpha (8–10 Hz) components [60]. While the alpha peak in young adults is usually observed at 10 Hz (e.g., [61]), a slowing of the alpha peak at 8–9 Hz can be observed with age [62]. Moreover, recent results point to a more different modulation of the alpha peak across brain areas in response to task demands in young compared to older adults, suggesting an age-related reduction of top-down control of sensory processing [63]. Finally, alpha synchronization in older adults is greater in frontal than in parietal regions [57], while an age-related shift of the alpha rhythm sources in the anterior-to-posterior direction has been observed [60,64]. Overall, these findings point to a different frequency-specific spatiotemporal modulation of the alpha activity during wakefulness in the elderly. If the SO process implies a transition between two distinct functional roles of the regional wake and sleep alpha activity, it is conceivable that age-related alterations in the waking alpha rhythm should be mirrored by (and probably functionally related with) age-related modifications of the regional alpha pattern during the wake-sleep transition. Starting from the observations of a slower alpha frequency and a modification of the distribution of alpha power and incidence from posterior to anterior regions during wakefulness in the elderly [65], it could be speculated that the generalized power reduction in the low alpha frequency bins during the SO may represent the disappearance of the such slower waking alpha peak, while the anterior post-SO increase in the high alpha bins represents part of the frontocentral synchronization process during sleep. However, the functional significance of such modification (i.e., the post-SO reduction of the 8–9 Hz power in the elderly) should be directly investigated in future studies. 

### 4.4. Sigma Activity

The sigma band represents the frequency range that characterizes sleep spindles [66], the typical electrophysiological hallmark of NREM sleep that is classically divided in two types: slow frontal (~11–13 Hz) and fast centroparietal (~13–15 Hz) spindles. Using different definitions of the wake-sleep transition, several studies described in young adults a strong growth of the sigma power after the SO [24,26,27] with clear centroparietal maxima [26]. Here, the EEG pattern in the sigma range described in older adults appears only partially similar to the one previously observed in young adults. On the one hand, we observed a post-SO increase in sigma activity, mainly localized in the centroparietal regions at 13–14 Hz and in frontal derivations at 12 Hz, and progressively greater during the initial part of the 1st NREM sleep cycle. These findings are consistent with the view of a post-SO increase of both fast centroparietal and slow frontal sleep spindles. On the other hand, several peculiarities should be underlined. First, while in young adults, sigma activity exhibits one of the most intense post-SO power increases [26], it shows only a slight enhancement in our sample of older subjects. Moreover, the sigma increase in young adults is clearly stronger in the centroparietal areas at 13–14 Hz than in the anterior slow sigma [26], while older subjects in the present study do not show a clear regional frequency-specific maximum enhancement (i.e., the frontal slow sigma increase appears as intense as the centroparietal fast-sigma enhancement). Finally, we found a post-SO sigma reduction in frontotemporal regions at 14–15 Hz, greater and more diffuse with increased frequency, that was not observed in young adults [26]. NREM sigma activity decrease in middle-aged and older adults compared to younger ones [6,7,8,66]. Consistently, sleep spindles exhibited reduced number/density, peak, duration, and amplitude with increasing age [11,13,66]. The maximal reduction of sigma and spindle density and amplitude was observed in frontal regions [6,11,13,66], while the decrease in duration was greater in posterior regions [13]. Albeit, we did not directly compare sigma activity in young and older adults, our results are consistent with the evidence of reduced sigma/spindle activity in physiological aging. The description of spatiotemporal spindles features during the SO in the elderly will be crucial to better characterize this phenomenon [27]. It should be considered that healthy elderly individuals are mainly characterized by a slowing of the EEG rhythms [67,68]. In this view, the post-SO frontotemporal reduction in the high-sigma range observed in our study may actually represent a decrease in a slower beta rhythm, consistent with the pattern observed in the beta frequency bins (see below).

### 4.5. Beta Activity

Bins in the beta range exhibited a generalized power decrease after SO, progressively stronger during the first NREM sleep cycle (with only a slight central increase in close proximity to the beginning of REM sleep). Such a finding substantially replicates the pattern previously observed in young adults [24,25,26,69,70], pointing to a reduction of arousal after SO, being the EEG high frequencies that are considered as markers of motor/cognitive activation [71,72,73,74].

## 5. Conclusions

Healthy aging is characterized by pronounced changes in the EEG pattern of wakefulness [75] and sleep [5]. In the present study, we described for the first time the spatiotemporal modifications that characterize the wake-sleep transition in a group of older adults. We found several age-related specificities, mainly pointing to a different topographical regulation of alpha and sigma frequencies in the elderly. Moreover, the direct comparison with a group of young adults highlighted in the elderly a reduction of SWA and delta/beta ratio during SO, suggesting a reduced homeostatic dynamic and a greater arousal level during the wake-sleep transition. 

These findings denote the importance of a better knowledge of the aging-related EEG features of the SO process. Such an approach can help to provide a clear electrophysiological basis for the longer SO latency and greater sleep fragmentation observed in the elderly [5] and could represent the basis for future intervention strategies. Indeed, several studies suggest that transcranial current stimulation methods may affect the human EEG dynamics of the wake-sleep transition to enhance sleep propensity [23,76,77,78]. Moreover, non-invasive brain stimulation techniques have been also used to modulate NREM sleep EEG oscillations with beneficial effects on cognitive performance [79,80], and their efficiency in healthy and pathological aging has been recently assessed with heterogeneous but promising results [81,82]. In this view, the description of the specific spatiotemporal EEG dynamics during SO in the elderly could provide evidence of the possible electrophysiological targets of intervention to reduce the duration of the wake-sleep transition and the sleep fragmentation in this population. 

It should be underlined that we performed retrospective analyses on data not directly collected with the aim to assess age-related changes in the SO EEG features. It is worth noting that we did not perform an adaptation night in the sleep laboratory because of the frequent difficulties reported by older adults to change daily habits and sleep outside their homes for consecutive nights. Since our young adults’ sample [26] underwent an adaptation night before the baseline recordings, we cannot exclude that some differences in the SO electrophysiology (and in particular, the differences between young and older adults in relative delta power and delta/beta ratio directly observed comparing these age groups) may be associated with the presence of a “first night effect” (FNE). This possible confounding factor should be addressed in future studies. However, the quantitative study of the FNE pointed out that NREM sleep of the first night is characterized by a significant and paradoxical increase in the power of slow frequencies (1–5 Hz) without regional differences [83]. This means that the reported differences concerning SWA are unlikely attributable to the FNE. 

Finally, since we did not perform a direct comparison between age groups concerning theta, alpha, and sigma frequency ranges, we cannot clarify if the peculiar topographical pattern observed in these bands exhibited by the elderly actually mirrors a regional difference in the EEG power density between young and older adults. 

Beyond replicating our findings and confirming them with longitudinal protocols, future studies should be aimed to characterize the aging-related modifications in the electrophysiology of the SO process considering the spatiotemporal dynamics of other crucial features [23]: the genuine oscillatory rhythms, the cortical sources, the specific SO-related EEG events (i.e., slow waves and sleep spindles), and measures of cortical connectivity.

## Figures and Tables

**Table 1:** Polysomnographic measures. Means and standard errors (SE) of the PSG variables in 36 healthy elderly subjects and 40 healthy young adults. SWS = slow wave sleep; REM = rapid eye movement; WASO = wake after sleep onset; TST = total sleep time; TBT = total bed time; SEI = Sleep Efficiency Index. Results of the comparisons (*t*-tests) between the two age groups are also reported. Asterisks index significant differences.

	Older Adults Mean ± SE	Young Adults Mean ± SE	*T*	*p*
Stage 1 latency (min)	14.9 ± 2.7	6.6 ± 0.9	3.9	* 0.003
Stage 2 latency (min)	15.7 ± 2.6	11.1 ± 1.8	1.4	0.20
Stage 1 (%)	5.0 ± 0.5	6.3 ± 0.5	−1.9	0.06
Stage 2 (%)	77.1 ± 0.9	59.3 ± 1.1	12.6	* <0.00001
SWS (%)	0.8 ± 0.2	10.3 ± 1.0	−8.8	* <0.00001
REM (%)	17.1 ± 0.8	24.1 ± 0.8	−6.2	* <0.00001
WASO (min)	72.2 ± 7.2	26.1 ± 3.0	6.1	* <0.00001
Awakenings (#)	18.9 ± 1.4	28.6 ± 1.7	−4.3	* 0.00005
Arousals (#)	19.9 ± 1.8	35.4 ± 2.8	−4.5	* 0.00002
TST (min)	298.2 ± 9.1	441.4 ± 6.1	−13.3	* <0.00001
TBT (min)	385.7 ± 7.4	484.8 ± 10.1	−7.8	* <0.00001
SEI % (TST/TBT)	77.5 ± 2.0	91.7 ± 1.1	−6.4	* <0.00001

## Data Availability

The data presented in this study are available on request to the corresponding author.
